# Observations on Scurvy, Especially with Reference to Its Pathological Anatomy

**Published:** 1845-07

**Authors:** 


### ART. XIII.

*Beobachtungen über den Scorbut, vorzüglich in pathologisch-anatomischer Beziehung.* Von G. v. SAMSON-HIMMELSTIERN.—*Berlin,* 1843.

*Observations on Scurvy, especially with reference to its Pathological Anatomy.* By DR. G. v. SAMSON-HIMMELSTIERN, Chief Physician in the Alexander-Cadet-Corps at Brest-Litowski.—*Berlin,* 1843. 8vo, pp. 156.

SINCE 1795 or 96, scurvy may be said to have ceased to exist in the British navy; and the improvements in horticulture and general hygiene have rendered it almost unknown to the civil practitioner; but to our shame it may be stated, that our prisons occasionally afford instances of it, and that the British mercantile marine is still disgraced by its frequent occurrence. The latter stigma however, it may be hoped, will not exist much longer, in consequence of a late enactment, which compels a proper supply of the necessary preventives of the disease, to the crews of all vessels proceeding on long voyages: but the fact, that in this country—which may boast of having been the first to prove, on a large scale, how easily this disease may be prevented, and cured,—it should still continue to exist in any public institution, and in merchant vessels, will tend to diminish the surprise that the appearance of this work of Dr. Himmelstiern's would otherwise have been calculated to produce. By it we find that scurvy still prevails to a lamentable extent in the imperial naval hospitals and depôts of Russia, although we believe it does not occur in her fleets when at sea. That this disease should be rife among the lower classes of people in the northern parts of that empire is what might readily be supposed, when we call to mind its former prevalence and great fatality in this country, so late as the commencement of the 18th century, but that it should, we might almost say be *allowed,* to exist to so fatal an extent in the Russian naval hospitals, as to afford the materials for such a work as this, is really remarkable. One of two causes must be assigned for such a difference in the sanitary condition of the inmates of a Russian and a British naval hospital; either the means found by the experience of fifty years to be so efficacious for the prevention of the disease in this country for some reason lose their efficacy in Russia, or they are not employed at all. We have no knowledge of the regulations of the Russian service in this matter, but it would appear, from the intrinsic evidence afforded by the work of Dr. Himmelstiern, that the latter is in all probability the true state of the case.

If his conjectures as to the cause and nature of scurvy represent, as we are inclined to believe they do, the opinions entertained generally in the Russian naval service on this subject, the continued prevalence of this scourge there may reasonably be attributed at least as much to the ignorance which prevails as to its true nature and the means for its prevention, as to any peculiarity of climate or constitution.

The object of Dr. Himmelstiern, in the present work, has been more the elucidation of the pathology of scurvy than its etiology or treatment; but as he does shortly state his views on the former subject at the end of the book, and frequently refers incidentally to its treatment in the body of it, we shall not be unjust in considering it, so far as it goes, as a summary of his notions on both these points.

His opinions as to the cause of scurvy are, that it arises:

1st. From a terrestrial miasm, which prevails chiefly in the spring, and also from a miasm peculiar to *hospitals,* prisons, and ships.

2dly. To a prolonged suppression of the cutaneous secretion.*

3dly. He enumerates innutritious food, and depressing mental impressions, as among the circumstances which aid the influence of the above causes.

He also states that the proximate cause of the disease may be considered to consist in a broken down condition of the blood, an incipient death of it, and to an obstructed innervation of the vessels. The author's treatment seems to be in accordance with his notions of the etiology and nature of

* He strengthens this argument by adducing, among others, Gluge's experiments on frogs and puppies, in which he had prevented the cutaneous transpiration by the application of a turpentine varnish, and in all these instances found, after death, a remarkable fluidity of the blood, and the author believes that a similar condition of the blood is induced in scurvy by the dry and unperspirable condition of the skin in that disease. See also our review of M. Fourcault's work in the present Number.

the disease—vague and theoretical; and we are thus able, perhaps, to surmise one reason of its greater prevalence in the naval service to which he belongs, than in our own.

Leaving these considerations, however, out of the question, his work will be found to contain a tolerably copious summary of all that has been described, with regard to the pathological anatomy of the affection, than which perhaps few points connected with the disease have been less satisfactorily treated. The older writers, who had abundant opportunities of making investigations on the subject, have so obviously allowed their imaginations to bring what they observed into accordance with their various theories of the nature of the disease, that little of any value can be gathered from them; and later ones have apparently attended less to this part of the subject of scurvy than to the more immediately useful consideration relating to its prevention and cure. Animal chemistry appears to be the source from which any additional information of importance is to be looked for. And a correct account of the changes induced in the blood by the influences which produce the scorbutic condition, is yet a desideratum of very great importance.

Dr. Himmelstiern arranges his subject according to the alterations produced by scurvy in

I. *The tissues.* 1. The integuments. 2. The cellular tissue, or fat. 3. The muscles. 4. The fibrous tissues. 5. The bones. 6. The mucous membranes. 7. The serous membranes. 8. The glands. 9. The nervous tissue. 10. The vascular system.

II. *The organs.* 1. The lungs. 2. The spleen. 3. The liver, kidneys, &c.

III. *The fluids.* 1. The blood. 2. The urine.

The principal interest of the present work consists in the minuteness and apparent accuracy with which the author describes the pathological changes he has observed. His descriptions agree very closely with what we have observed ourselves, and we do not perceive that he offers anything very novel or original, even in his theories, some of which date from Cullen and the older pathologists; while he has fallen, very extensively as it appears to us, like many of his predecessors, into the error of confounding the morbid changes, due properly to other affections, which have happened to be concomitant with scurvy, with those belonging simply to that affection. This error is committed especially with regard to some forms of cutaneous eruption, which, although merely modified by the scorbutic diathesis, he is inclined to consider as peculiar to that affection.

He describes the true scorbutic affections of the skin with great accuracy and minuteness. Under the head of changes in the cellular tissue, he describes the effusions as they occur in the cellular and fatty tissues, under the skin, and among the muscles, and under this head enumerates four sorts of effusion.

"1. Effusion of serous or sanguinolent fluid in the subcutaneous cellular tissue, which may be distinguished from the œdema arising from other causes, by its greater hardness—which approaches that of wax, and by the length of time it retains the impression of the finger, and also by the situation of the parts in which it usually commences, which are not always the most dependent portions of the limbs. The swelling depends upon the effusion of a fibrinous and serous fluid more or less tinged with the colouring part of the blood into the subcutaneous cellular tissue.

"2. Circumscribed swellings in various parts, as the consequence of slight injuries or of violent muscular exertion.

"3. The changes which occur in the adipose tissues.

"4. The more important of the effusions caused by scurvy are those which occur in the intermuscular and subfibrous cellular tissue. These consist either of a sanguinolent fluid, or of thin layers of coagulated blood situated in the interstices between the muscles or on their immediate surface, and which are readily removed, or there are bloody-coloured layers of a gelatinous consistence, marked with streaks of a yellowish white, resembling sometimes blood coagula or the fibrinous clots met with in the heart. The muscles bounding these effusions, are infiltrated with blood, and their substance is weakened in consequence, the fibrous tissue being scarcely apparent. Lastly, we find these intermuscular deposits in another form, in which they exhibit a higher degree of organization than the above. Deposits of this kind occur in the form of layers of from a quarter of a line to a line in thickness, and are composed apparently of *fibrine* of a bright or yellowish red colour, firm and elastic and affording no fluid on pressure. They are true false membranes, and are quite distinct from the surrounding muscles to which they firmly adhere. This kind of deposit has been termed 'scorbutic formation,' by Dr. W. v. Samson, in an unpublished work. They occur not only under the fascia and between the muscles of the extremities, but also between the broad expansions of the abdominal and pectoral muscles. The stiffness of the joints and especially of the knees, appears to be caused by the firm consistence of these effusions; and the muscular pains, are probably caused by the position they occupy and by their pressure, and to them also may in part be attributed the hardness of the limbs. They exhibit the physical properties of fibrine, from which all fluid has been removed, and as it occurs in false membranes." (p. 25.)

In the fibrous tissues and bones the principal appearances adverted to are the occurrence of effusions in the external surface of the periosteum and between that membrane and the bone (scorbutic nodes.) With regard to these nodes it is truly remarked, that they differ in several respects from those arising in consequence of syphilis. "They are formed much more rapidly and are attended with marked nocturnal exacerbation of pain."

We have referred rather at length to the author's description of this last class of effusions, or those which occur between the muscles and beneath the periosteum, for the purpose of remarking, that it serves to strengthen an opinion we have long been inclined to hold, that the effusions which occur in scurvy are not caused, as is commonly supposed, simply by extravasation of blood.

These effusions, or, as they are styled by Dr. Samson, "*scorbutic formations,*" and also those which cause the swelling of the gums, can, we think, be more properly considered as effusions of organizable fibrine, than as blood coagula, admitting of vascular organization. Upon injection these effusions will be found to be very richly furnished with capillary vessels, which in their character and mode of distribution are so perfectly similar to the newly-formed capillaries in other recently organized adventitious tissues, that they cannot fail to be recognized as of the same nature.

This point is alluded to in a paper by Mr. Dalrymple, in the 'Medico-Chirurgical Transactions,' who has subjoined a figure representing the distribution of the capillary vessels, injected in a deposit of the nature we are describing, which was situated beneath the periosteum of the liver in a man who died of scurvy. It might be considered that the effusion in this case depended upon simple periostitis or osteitis, in a scorbutic subject; but the nodes during life were so precisely of the character of those frequently met with in scorbutic patients, numerous instances of which have more recently fallen under our observation, that there is no reason to suppose that they depended on any other cause than scurvy. We have in many instances seen this sort of node in cases where no suspicion of syphilis was admissible, and in whom no mercury nor any previous affection of any kind could be considered their cause. Besides this, we have had an opportunity of injecting a limb which was the seat of extensive scorbutic effusions, and these were found even in the interior of the knee-joint. The solid material in the latter situation was partly adherent to the synovial surface and partly loose. The former portions were all more or less injected, the latter not; but in both instances, the masses on examination by the microscope exhibited numerous corpuscles, advancing, according to the opinion of Mr. Dalrymple who examined them, to the stage which precedes organization in fibrinous effusions.

Leaving, however, these artificial proofs, if they may be so considered, out of the question, let us consider the nature and extent of the peculiar condition of the gums in scurvy, and it will probably be allowed that this hypertrophy, as it may in reality be called, can only be caused by the deposit in the tissue of a plastic material. The swollen gums are firm, and evidently organized. They are sensible, and bleed when wounded, and the swelling admits of the most rapid absorption, as in fact do all solid scorbutic effusions. The remarkably circumscribed extent also of the affection of the gums precludes the probability of the swelling being caused by simple effusion of blood or other fluid into their tissue, but would apparently serve to strengthen the supposition that the additional matter is of a solid nature. These considerations, and others which it would require too much space to adduce, lead us to conclude that most of the effusions which occur in scurvy are not really of blood as such, but that they consist of a plastic fibrinous matter coloured with blood.

The error of confounding various pathological changes, modified by the scorbutic diathesis, with those properly belonging to that disease alone, is very evident in the section which treats of the affections of serous membranes. The morbid changes observed in this tissue are referred principally to the pleura and pericardium, and appear to have been met with very frequently in a peculiar epidemic which occurred in and about Moscow chiefly in 1840. This epidemic, whatever was its true nature, was characterized by a remarkable predominance of pericardiac and pleuritic effusions; for out of 66 cases dissected by Dr. Karawajew, Pericarditis occurred 30 times; Pleurisy, 22; Pericarditis and Pleurisy, 6; Peritonitis, 7; Arachnitis, once. But it does not appear at all clear that these effusions were caused by scurvy, though it is evident, from the modified appearances they presented, chiefly as regards their colour, that those who were thus affected laboured under a scorbutic diathesis. The operation of *paracentesis* of the pericardium appears to have been several times successful, and many cases are given in detail.

We have seen many cases of death from pure scurvy, but have not observed any effusions similar to those described by Dr. Himmelstiern in the various serous cavities.

The only affection of the pulmonary tissue which the author appears to consider, as peculiar to scurvy, is a more or less extensive infiltration of it with black fluid blood, effused in equal quantity on the anterior and posterior parts, and which is attended with a remarkable friability and dark colour of the whole tissue. The sound on percussion, he says, is scarcely affected in this condition of the lung, but auscultation detects a rough respiratory murmur. He has also occasionally observed a blowing sound, which he attributes to the arteries. Part of the dyspnea, which is so distressing in scurvy, he believes to depend upon this congestive state of the lungs.

The *spleen* was formerly thought to be usually much affected in scurvy, but it is clear that for this opinion there are no sufficient grounds. A morbid appearance of this organ is described, however, as having occurred to the observation of the author and others; viz., the presence of wedge-shaped bodies on its surface, and which he considers as analogous to the superficial hepatization of the lungs, and to be caused either by fibrine deposited, in consequence of the morbid condition of the blood, or to a process connected with phlebitis—a point which he thinks might be determined by micro-chemical examination.

Nothing remarkable appears to have been met with in the other solids. The author considers a nodulated and contracted condition of the liver (cirrhosis) to be an occasional *cause* of scurvy, which in such cases is attended with great enlargement of the spleen.

The condition of the blood in scurvy has been so differently described by various authors, and their descriptions have been so evidently drawn rather from the peculiar tenets of the pathological school to which the writers have belonged, than from nature, that it would be impossible to gain correct notions on the subject from them, and especially from any of the older writers, by whom in turn all imaginable qualities have been attributed to the scorbutic blood. Dr. Himmelstiern thinks that some of these discrepancies may be reconciled, by supposing that the condition of the blood may vary materially, according to the stage of the disease at which it is observed, or according to the affections with which it may be complicated. He considers that in consequence of the separation of the fibrinous element in the various effusions, the blood becomes impoverished in the latter stages of the disease, and assumes a dissolved, incoagulable character.

We would only remark upon this supposition, that in several examinations of the blood of persons labouring under scurvy in a severe form, we have uniformly found the proportion of fibrine and salts and water to exceed the average, and that of the hematosine to fall below it. Blood drawn in scurvy has, in all the cases in which we have tried the experiment, formed a small firm clot, usually buffed and sometimes cupped; but to assert that this will be found universal, would be so directly to contradict the observations of many others, that we cannot venture to do it.

As the author does not professedly treat of the prophylaxis, or cure of the disease, we can only gather from incidental notices in the accounts of some cases, which he has subjoined at the end of the book, what his principles and practice are in these respects. Upon these we have already expressed our opinion, and we cannot but regret that he has not profited more in these respects from his study of English authors, with whose works his frequent references to them prove him to be well acquainted. The experience of half a century has, at all events, shown that whatever may be our real knowledge of the pathology of scurvy, we have in this country known how to cure, and, what is more, to prevent it; and, if we might so far presume, we would suggest to Dr. Himmelstiern that labour and talent equal to that which he has displayed in this interesting work on the Pathology of Scurvy, if devoted now to its Etiology, prevention and cure, would doubtless meet with commensurate success; and that from his endeavours would accrue the same advantages to the Russian naval service as have resulted from the sagacious example of Cook, and the scientific exertions of Lind, of Trotter, and of Blane, to that of Great Britain.

---

